# Evidence-Based Selection on the Appropriate FIT Cut-Off Point in CRC Screening Programs in the COVID Pandemic

**DOI:** 10.3389/fmed.2021.712040

**Published:** 2021-07-27

**Authors:** Rocío Aznar-Gimeno, Patricia Carrera-Lasfuentes, Rafael del-Hoyo-Alonso, Manuel Doblaré, Ángel Lanas

**Affiliations:** ^1^Department of Big Data and Cognitive Systems, Instituto Tecnológico de Aragón, ITAINNOVA, Zaragoza, Spain; ^2^Biomedical Research Networking Center in Hepatic and Digestive Diseases (CIBERehd), Madrid, Spain; ^3^Aragón Health Research Institute (IIS Aragón), Zaragoza, Spain; ^4^Aragón Institute of Engineering Research (I3A), Zaragoza, Spain; ^5^Department of Mechanical Engineering, University of Zaragoza, Zaragoza, Spain; ^6^Biomedical Research Networking Center in Bioengineering, Biomaterials and Nanomedicine (CIBERbbn), Madrid, Spain; ^7^Department of Medicine, Psychiatry and Dermatology, University of Zaragoza, Zaragoza, Spain; ^8^Service of Digestive Diseases, University Clinic Hospital, Zaragoza, Spain

**Keywords:** colorectal cancer, screening fecal-immunological test, decision-making, colonoscopy, adenomas

## Abstract

**Background:** The COVID pandemic has forced the closure of many colorectal cancer (CRC) screening programs. Resuming these programs is a priority, but fewer colonoscopies may be available. We developed an evidence-based tool for decision-making in CRC screening programs, based on a fecal hemoglobin immunological test (FIT), to optimize the strategy for screening a population for CRC.

**Methods:** We retrospectively analyzed data collected at a regional CRC screening program between February/2014 and November/2018. We investigated two different scenarios: not modifying vs. modifying the FIT cut-off value. We estimated program outcomes in the two scenarios by evaluating the numbers of cancers and adenomas missed or not diagnosed in due time (delayed).

**Results:** The current FIT cut-off (20-μg hemoglobin/g feces) led to 6,606 colonoscopies per 100,000 people invited annually. Without modifying this FIT cut-off value, when the optimal number of individuals invited for colonoscopies was reduced by 10–40%, a high number of CRCs and high-risk adenomas (34–135 and 73–288/100.000-people invited, respectively) will be undetected every year. When the FIT cut-off value was increased to where the colonoscopy demand matched the colonoscopy availability, the number of missed lesions per year was remarkably reduced (9–36 and 29–145/100.000 people, respectively). Moreover, the unmodified FIT scenario outcome was improved by prioritizing the selection process based on sex (males) and age, rather than randomly reducing the number invited.

**Conclusions:** Assuming a mismatch between the availability and demand for annual colonoscopies, increasing the FIT cut-off point was more effective than randomly reducing the number of people invited. Using specific risk factors to prioritize access to colonoscopies should be also considered.

## Introduction

The COVID pandemic has forced the closure of many colorectal cancer (CRC) screening programs around the world. Most endoscopy units have limited their activity to urgent procedures or to patients with a high suspicion of gastrointestinal cancer (1). Furthermore, resuming normal endoscopic activity has been slow and the number of procedures per room/day has been reduced. This situation poses a challenge for CRC screening programs, because in most cases, endoscopic units will not be able to resume the same activity levels practiced before the pandemic (2, 3).

The key factors that determine the level of activity in CRC screening programs are the number of endoscopic procedures available in the endoscopic units, the number of people invited, and the type of test used. When a fecal occult blood test is used, the cut-off point determines which patients will undergo colonoscopy. Thus, the cut-off point is the most important factor for selecting which people are invited, and it is directly related to the other factors. It is difficult to determine the cut-off point, because raising this value increases the risk of missing a number of high-risk lesions or cancers (4). On the other hand, maintaining the current cut-off values with insufficient colonoscopy availability will delay the inclusion of patients in screening programs, due to the current restrictions, which will cause an undetermined delay in the diagnosis of high-risk lesions and cancer (5).

In this study, we analyzed, from a temporal perspective, the CRC screening program outcomes for the Aragón region (Spain). We aimed to design a general methodology and provide data that might facilitate evidence-based decisions by managers and health authorities on how to reinitiate CRC screening. Our approach was to optimize the strategy for screening a population for CRC, with the objective of minimizing the expected number of missed lesions and delayed diagnoses.

## Materials and Methods

### Study Population

We evaluated data on individuals that participated in the CRC screening program in the Aragon region (Spain) between February 2014 (the start of the population-level program) and November 2018. Individuals invited to the program were at medium risk, aged 60–70 years, had no family history of CRC, had no previous colonoscopy in the previous 5 years, and had no known colonic diseases, colectomy, or irreversible terminal diseases. The screening program was originally planned (before the pandemic) to extend to patients aged 50–59 years, within the universal health system. However, those individuals were excluded from the first round of invitations to maximize the benefits of the program, because endoscopic units were already busy coping with symptomatic patients.

### Fecal Immunochemical Test, Colonoscopy, and Lesions

Invited individuals that agreed to participate in the program had to be asymptomatic. They underwent selection, based on a fecal immunochemical test (FIT) (FOB Gold^®^ SENTiFIT; Sysmex-Sentinel CH. SpA, Barcelona,Spain). The cut-off value used during the program was 20 μg hemoglobin/g feces, which is the standard used in Spain and most European countries (6).

Patients with a negative FIT result were temporarily excluded from the program for 2 years. Otherwise, the patient was invited to undergo a colonoscopy. When the colonoscopy did not show any lesion, the patient was temporarily excluded from the program for 10 years. However, when either adenomas or cancers were detected, the patient was permanently excluded from the program and transferred to either the gastrointestinal outpatients or ward for follow-up or treatment.

### Colonoscopy, Histologic Examination, and Definitions

Colonoscopies were performed by experienced gastroenterologists from different units of Digestive Diseases Services in the community. The quality standards were established by the European Society of Gastrointestinal Endoscopy (7, 8). Any polypoid lesion detected in the procedure was removed and classified by an experienced pathologist. The classes were established by the Spanish Network of Cancer Screening Programs (http://www.cribadocancer.es/), based on the European guidelines for quality assurance in CRC screening and diagnosis (9).

The study end points were the lesions, which were classified according to the following grades:

1. “Low-risk adenomas,” defined as 1–2 tubular adenomas <1 cm with low grade dysplasia.
2. “Intermediate-risk adenomas,” defined as ≥3 adenomas, or adenomas ≥1 cm, with a villous histology or high-grade dysplasia.
3. “High-risk adenomas,” defined as ≥10 adenomas or adenomas ≥2 cm (mutually exclusive with intermediate-risk adenoma > 1 cm)
4. “Colorectal cancer,” defined as any invasive cancer of the colorectal mucosa that reached the submucosa, regardless of the subsequent stage in the TNM classification^1^.

### Data Curation

We collected data on program participants from data recorded in the program database by different staff members and data uploaded from external databases. Endoscopists of the different centers recorded the data on endoscopic findings. The medical staff in the CRC program recorded data related to the patients, the histological analysis of specimens obtained from endoscopy or surgery, and the treatment given after cancer detection. Therefore, our first step was to perform an extensive evaluation of the quality of the information stored and to carry out a curation process to procure a clean source of information. Detailed information on this process can be found elsewhere (10). Once the data curation was performed, the resulting database could be used for analyses of any particular subpopulation, defined by sex, age, medical district, province, etc.

The methodology used to aid decision-making was based on the relationship between the number of people invited to the program, the number of annual colonoscopies available, and the established FIT cut-off point. Each of these values could be estimated, based on the other two values. Additionally, we analyzed data on the population screened between 2014 and 2018 to estimate the expected number of lesions missed or diagnoses delayed, based on the projected parameters of the three main variables (number of people invited, number of annual colonoscopies performed, and the established cut-off point).

### Hypotheses and Analysis

In this study, we assumed that the behavior of the entire target population was homogeneous over time, which included the following corollaries:

1. The number of tests performed (with respect to the number of patients invited, i.e., rate of participation) remained constant over time.
2. The distribution of the stool blood concentration in the population or subpopulation did not change with time; accordingly, the rate of positive tests with respect to the population invited also remained constant over time.
3. The percentage of lesions and of the relative distribution of the risk characteristics (i.e., low risk, medium risk, high risk, and cancer) in the participating population did not change over time.

### Scenarios

**Figure 1:**
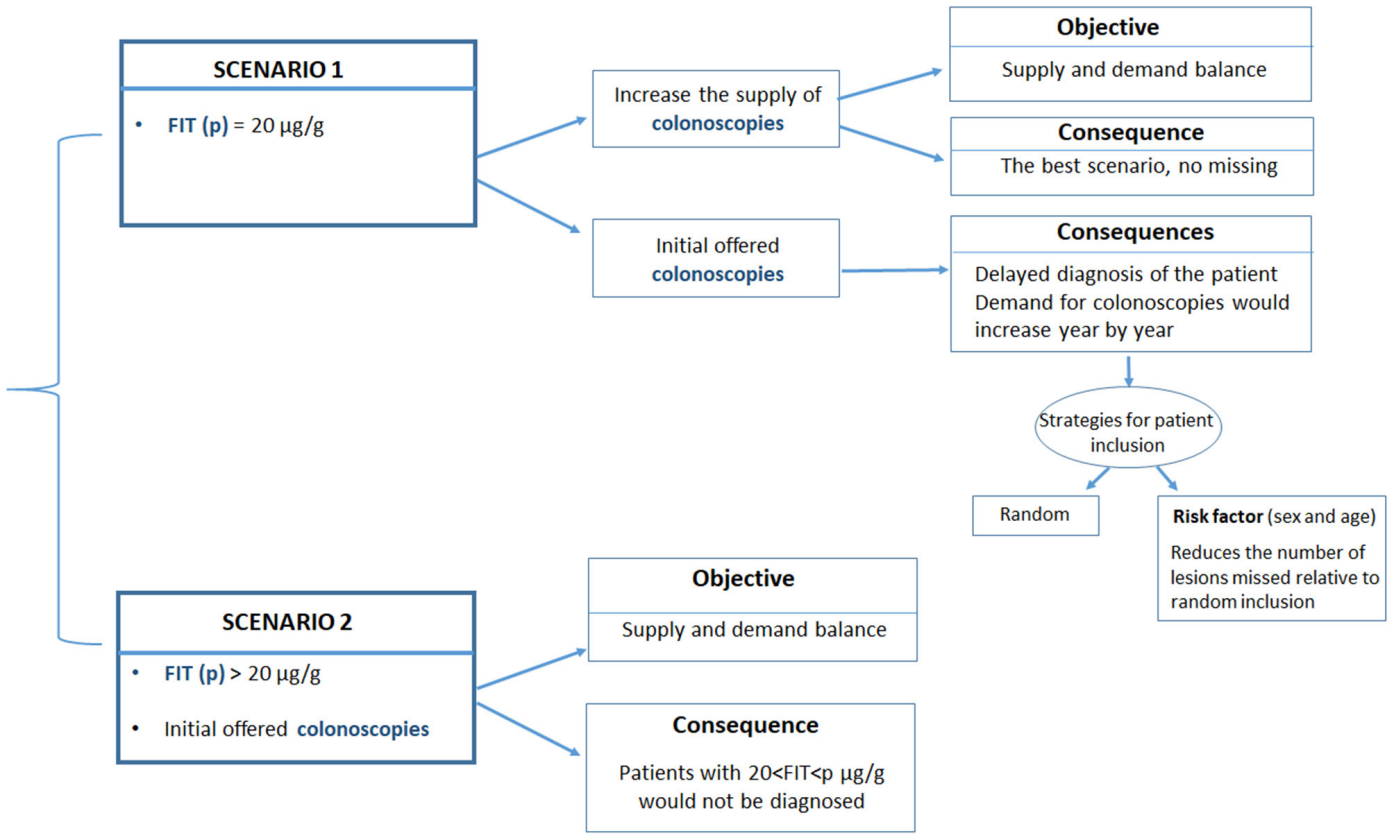
Description of the two scenarios analyzed in the current study. Scenario 1 maintains the current FIT cut-off point (20 μg hemoglobin/g feces), regardless of the annual demand for colonoscopies. Scenario 2 increases the current FIT cut-off point to match the number of colonoscopies to the expected number of positive cases for a given number of invitations.

We analyzed two potential scenarios (Figure 1):

1. The current cut-off point was 20 μg hemoglobin/g feces, regardless of the annual demand for colonoscopies.
2. The current cut-off point was increased to match the number of individuals invited for colonoscopies; here, the demand was derived from the expected number of positive tests for a given number of invitations.

Scenario 1 was optimal, providing that sufficient colonoscopies were available to meet the yearly demand. When the demand could not be met, a certain percentage of individuals with potential neoplastic lesions will not be diagnosed, because they would not be invited to undergo a colonoscopy in the corresponding year. The estimated number of patients in need and the types of lesions that were not diagnosed was derived from the data obtained from the cohort of individuals screened between 2014 and 2018.

In Scenario 1, those individuals that were not eventually invited due to an insufficient colonoscopy offer (excess target group) were added to the new cohort invited the next year. Then, and in this scenario, we evaluated different strategies for selecting individuals for screening in the subsequent year. In the first “random strategy,” all patients (new invitations and the excess target group of previous year) are randomly selected according to the same criteria (Figure 1), that is, all patients assigned to a particular year have the same probability of undergoing a colonoscopy, independent of the year of their first invitation to the screening program. In a second “prioritization strategy,” the excess target group from the previous year would be attended first, and then individuals in the new yearly cohort would be attended. An extension to the prioritization strategy was to prioritize access to colonoscopy by considering risk factors other than the FIT, such as age or sex.

In this study, we analyzed the possibility of prioritizing invitations based on sex, because men were demonstrated to have a significantly higher risk of lesions than women. This approach aimed to show the importance of establishing a risk index that included both the stool blood concentration and other risk factors to minimize the number of high-grade lesions missed.

In Scenario 2, the cut-off point (cop) was set, based on the number of colonoscopies to be offered. Then, we estimated the rate and number of lesions that would be missed in that year. Patients with a FIT result below the new “cop” but a FIT result > 20 μg hemoglobin/g feces, will not be identified in this scenario.

The estimations for each of these two scenarios are presented considering 100,000 people invited. However, it would be easy to scale the results to any other number of invitations. After the pandemic, the number of colonoscopies actually performed has been drastically reduced. We considered different potential reductions in the offer of colonoscopies. Specifically, we analyzed 60–90% of the total number of colonoscopies that would have met the demand.

### Statistical Analysis

To demonstrate the potential effects of potential risk factors to be considered, we analyzed several subpopulations, defined by sex or age. Particularly, we compared the rate of positive tests in the invited population, the rate of lesion detection in colonoscopies, and the distribution of different risk levels among the lesions detected between different subpopulations. For this comparative analysis, we performed the compare proportions test. The level of significance in the study was set to 0.05. Analyses were performed with the R programming language (The R Foundation for statistical computing, Vienna, Austria) (11).

## Results

### Outcomes

The screening results are shown in Table 1 (2014–2018); 6.6% of the invited individuals had a positive test (FIT ≥ 20 μg hemoglobin/g feces). Among the individuals that underwent a colonoscopy, 52.8% had detectable lesions, and of these, 30.4% were classified as high-risk or had cancer. The different sex and age groups had different rates of lesion detection. The rates of positive FIT were significantly higher (*p* < 0.001) for men than women and higher for the older, compared to the younger age group. Also, the rates of detected neoplastic lesions were significantly different (*p* < 0.001) between men and women (0.61 vs. 0.40). The distribution of lesion types was also significantly different (*p* < 0.001) among the different combinations of sex and age (Table 1) The stool blood concentrations showed small differences over the different years (2014–2018), and we expected the curves to converge to a steady state level. Therefore, we assumed that those differences will not impact the qualitative trends, or the conclusions drawn from the analysis reported here. The number of colonoscopies performed was essentially linearly correlated with the population invited, assuming no important variations in the percentage of people that accepted the invitation, underwent the FIT, and had a positive result.

**Table 1:** Screening results for the study population and subpopulations.

	**Total**	**Male**	**Female**	**Age [60–65] y**	**Age [65–70] y**
Invited (*n*)	**146,811**	71,363	75,448	71,621	75,190
FIT Performed^a^	**76,452** **(52.1%)**	36,971 (51.8%)	39,481 (52.3%)	35,476 (49.5%)	40,976 (54.5%)
Positive FIT^b^	**9,699** **(6.6%)**	5,748 (8.1%)	3,951 (5.2%)	4,335 (6.1%)	5,364 (7.1%)
Colonoscopies^c^	**9,139** **(6.2%)**	5,433 (7.6%)	3,706 (4.9%)	4,051 (5.7%)	5,088 (6.8%)
Neoplastic lesions^d^	**4,823** **(52.8%)**	3,315 (61 %)	1,508 (40.7%)	2,126 (52.5%)	2,697 (53%)
Low risk lesions^e^	**1,399** **(29%)**	825 (24.9%)	574 (38.1%)	656 (30.9%)	743 (27.5%)
Medium risk lesions^e^	**1,959** **(40.6%)**	1,382 (41.7%)	577 (38.3%)	869 (40.9%)	1,090 (40.4%)
High risk lesions^e^	**1,003** **(20.8%)**	767 (23.1%)	236 (15.6%)	413 (19.4%)	590 (21.9%)
Cancer lesions^e^	**462** **(9.6%)**	341 (10.3%)	121 (8%)	188 (8.8%)	274 (10.2%)

a,b,c *Number and percentage calculated based on the total number of invitations*.

*^b^FIT cut-off point >20-μg hemoglobin/g feces*.

d *Number of people with lesions and percentage calculated based on the total number of people with lesions*.

e *Number of people with lesions and percentage calculated based on the total number of colonoscopies*.

**Figure 2:**
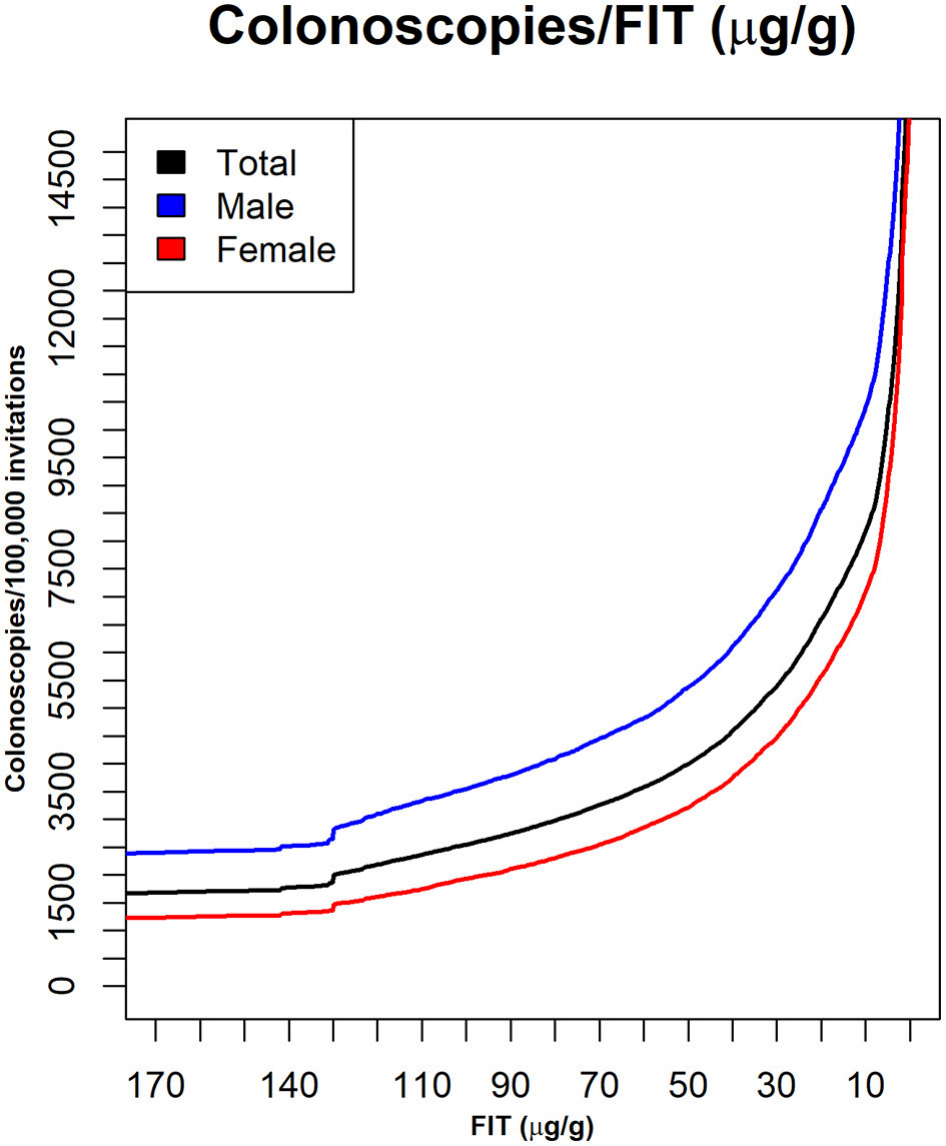
The numbers of colonoscopies required with different FIT cut-off values. Data are shown for the whole population and for sex groups. X axis: FIT (μg hemoglobin/g feces); Y axis: number of patients with a positive test, per 100,000 invitations.

**Figure 3:**
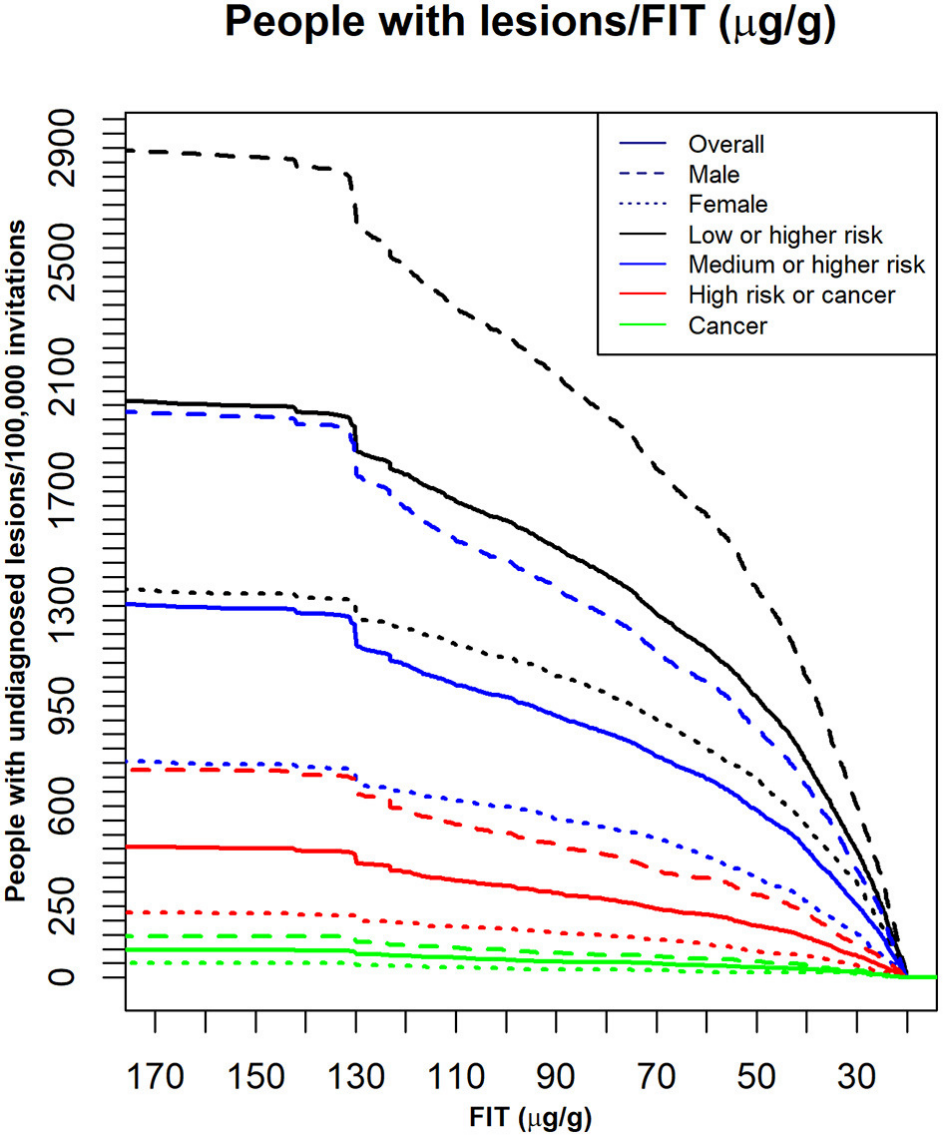
The number of individuals with undiagnosed lesions, based on the indicated FIT cut-off points. Dark lines: the whole population (Overall); Different colors: different lesion risk classes; dashed and dotted lines: different sex groups for each lesion risk class; X axis: FIT: (μg hemoglobin/g feces). Y axis: the number of people with undiagnosed lesions per 100,000 invitations.

Figure 2 shows the curves used to determine a cut-off point for matching a given availability of colonoscopies per 100,000 invitations. Figure 3 shows the number of people in each lesion risk class with undiagnosed lesions for each cut-off point considered Sex, as expected, was remarkably discriminating. With the same cut-off point, the number of men with undiagnosed high-risk lesions or cancer was very close to the number of women with medium- or higher-risk lesions. Age was also discriminating, but to a lesser extent (not shown).

### Scenario 1

With a constant cut-off point of 20 μg of hemoglobin/g feces, 6,606 colonoscopies would be required for each 100,000 people invited annually. However, if that number of colonoscopies was not available, and the cut-off point remained at 20 μg hemoglobin/g feces, the number of undiagnosed patients with positive tests would accumulate over the subsequent years, and individuals would not be invited in due time. We evaluated the following proportions of colonoscopy availability: 90–60% where 100% was 6,606 colonoscopies/100,000 people invited and required for the analyzed population.

Table 2 shows a comparison of the undiagnosed or delayed risk lesions for different colonoscopy availabilities in each scenario, and for each call criterion for the population of 1 year. We have explored an offer of colonoscopies of 90, 80, 70, and 60% of the 6,606 colonoscopies required for each 100,000 people invited annually when assuming the cut-off point of equilibrium (20 μg hemoglobin/g feces).

**Table 2:** Undiagnosed lesions per year considering scenarios 1 and 2 and the two call criteria in scenario 1.

		**Lesion risk class** ***N* (%)^a^**				
**% Of available colonoscopies**	**Scenarios**	**Low-risk**	**Medium-risk**	**High-risk**	**Cancer**	**Total lesions**
90%	**Scenario 1** Random call criterion	101 (10%)	141 (10%)	73 (10%)	34 (10%)	349 (10%)
	**Scenario 1** Prioritized call criterion	102 (10.13%)	103 (7.29%)	42 (5.82%)	22 (6.47%)	269 (7.71%)
	**Scenario 2**	97 (9.59%)	98 (6.93%)	29 (3.99%)	9 (2.67%)	233 (6.68%)
80%	**Scenario 1** Random call criterion	202 (20%)	283 (20%)	145 (20%)	67 (20%)	697 (20%)
	**Scenario 1** Prioritized call criterion	205 (20.25%)	206 (14.57%)	85 (11.63%)	44 (12.94%)	540 (15.48%)
	**Scenario 2**	203 (20.08%)	198 (14%)	62 (8.53%)	21 (6.23%)	484 (13.87%)
70%	**Scenario 1** Random call criterion	303 (30%)	424 (30%)	218 (30%)	101 (30%)	1,046 (30%)
	**Scenario 1** Prioritized call criterion	307 (30.39%)	309 (21.87%)	127 (17.45%)	65 (19.41%)	808 (23.16%)
	**Scenario 2**	298 (29.48%)	300 (21.22%)	108 (14.86%)	29 (8.6%)	735 (21.07%)
60%	**Scenario 1** Random call criterion	404 (40%)	568 (40%)	288 (40%)	135 (40%)	1,395 (40%)
	**Scenario 1** Prioritized call criterion	410 (40.51%)	412 (29.15%)	169 (23.26%)	87 (25.87%)	1,078 (30.9%)
	**Scenario 2**	398 (39.37%)	410 (29%)	145 (19.94%)	36 (10.68%)	989 (28.35%)

a *Percentage of non-diagnosed lesions per year with respect to the total number of estimated lesions of the same type in 100,000 invitations/year for the 2 scenarios and 2 call criteria considered*.

**Figure 4:**
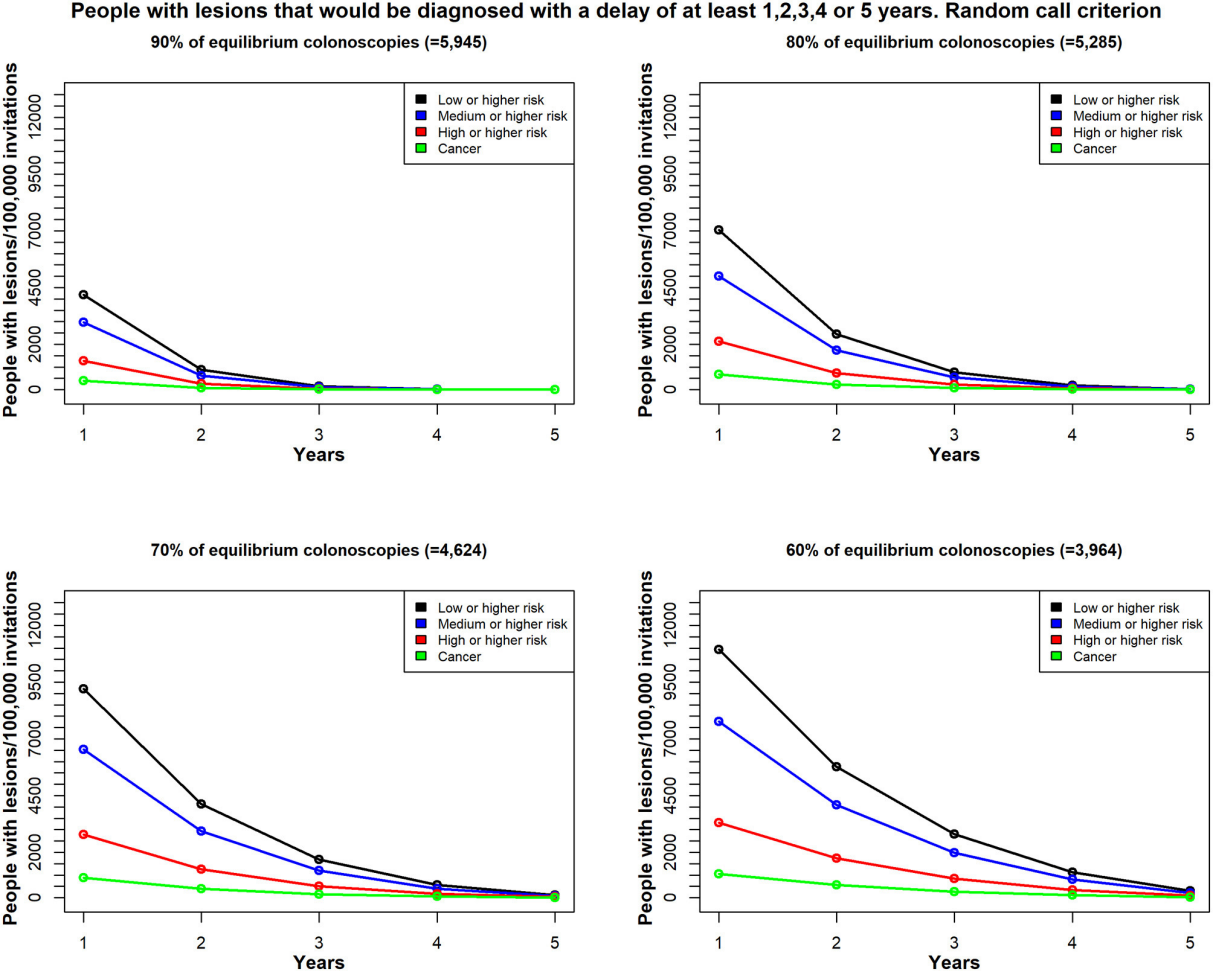
In Scenario 1, the time delay, due to unavailable colonoscopies, for a diagnosis among patients with colorectal lesions in each risk class; patients were re-invited, based on the random call criterion. Total demand for colonoscopies = 6,606/100,000 individuals per year; estimates reflect (*top left*) 90% availability (*n* = 5,945); (*top right*) 80% availability (*n* = 5,285); (*bottom left*) 70% availability (*n* = 4,624); and (*bottom right*) 60% availability (*n* = 3,964); Y-axis values reflect the estimated number of patients with lesions that would be diagnosed with a delay of at least 1 year, 2 years up to 5 years; X-axis values reflect the minimum delay (years).

Considering a period of 5 years, and assuming 100,000 invitations each year and the same colonoscopy availability in the whole 5 years period, Figure 4 shows the estimated number of patients with different types of colorectal lesions, whose diagnosis would be delayed by at least 1, 2, 3, 4, or 5 years, given the different scenarios of colonoscopy reduction. The graphs show that the lower the colonoscopy capacity the higher the number of lesions whose diagnosis will be delay for at least 1–5 years. For example, with 90% of equilibrium colonoscopy availability, no lesions will be delayed for more than 3 years (Figure 4). However, the risk of the lesions may progress with time, so the proportions of lesions with higher risk might increase with time, although this issue has not been addressed in this study.

**Figure 5:**
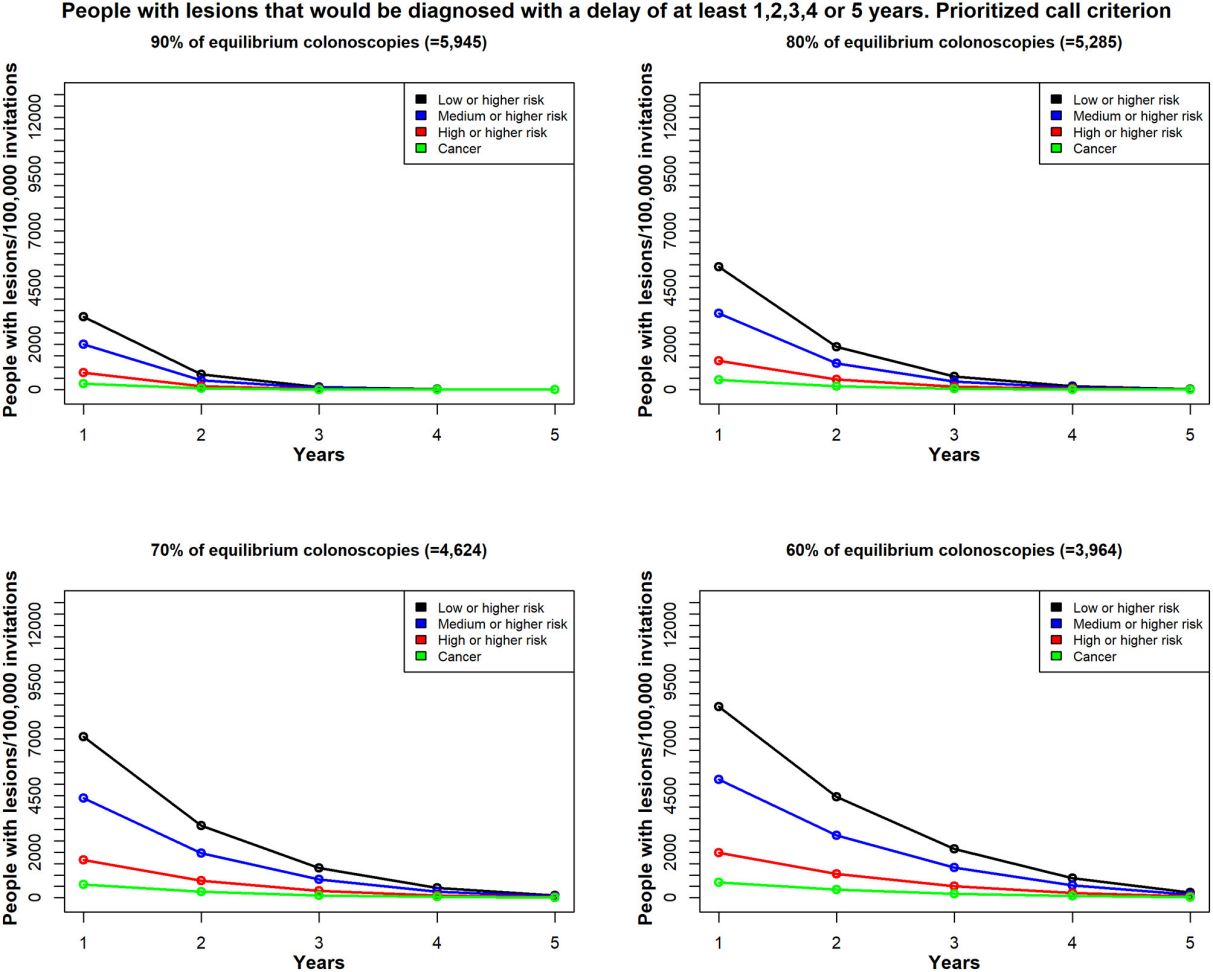
In Scenario 1, the time delay, due to unavailable colonoscopies, for a diagnosis among patients with colorectal lesions in each risk class; patients were re-invited, based on the prioritized call by sex (men) criterion. Total demand for colonoscopies = 6,606/100,000 individuals per year; estimates reflect (*top left*) 90% availability (*n* = 5,945); (*top right*) 80% availability (*n* = 5,285); (*bottom left*) 70% availability (*n* = 4,624); and (*bottom right*) 60% availability (*n* = 3,964); Y-axis values reflect the estimated number of patients with lesions that would be diagnosed with a delay of at least 1 year, 2 years up to 5 years; X-axis values reflect the minimum delay (years).

We found different results, depending on the selection strategy for re-inviting patients that could not undergo the timely colonoscopy (the excess target group). We compared two strategies; one employed the random call criterion, where the patients were invited at random (Figure 4). The second strategy employed the prioritized call criterion, which prioritized men for the invitations (Figure 5). We conducted this exercise to understand the effect of a potential risk factor, and we selected sex as a risk factor without regard to any possible policy decisions.

### Scenario 2

We found that the current cut-off point of 20 μg hemoglobin/g feces would be acceptable for 6,606 colonoscopies per 100,000 people invited annually, when there was no restriction on availability. However, this cut-off point would not meet the demand, when the colonoscopy availability was reduced to 90, 80, 70, or 60% of the demand. In those cases, to meet the demand, the FIT cut-off points would have to be raised to 25, 32, 40, and 51 μg hemoglobin/g feces, respectively (Figure 2). However, raising the cut-off point could result in missed diagnoses.

The estimated number of people with neoplastic lesions that would not be diagnosed when considering these cut-off points were extracted from Figure 3 and are shown in Table 2. For example, when 25 μg hemoglobin/g feces was the FIT cut-off point, 38 high-risk lesions or cancers and 9 cancers would not be diagnosed annually. These numbers represented, respectively, 3.6 and 2.7% of the lesions identified with the 20 μg hemoglobin/g feces cut-off in those risk classes.

## Discussion

The COVID-19 pandemic stopped or slowed many CRC screening programs. This interruption will have a great impact on the number of CRCs diagnosed and on the prognosis of newly diagnosed cases, which will, presumably, be diagnosed at an advanced stage (12–14). Resuming these programs is becoming a priority, but unfortunately, the new rules for preventing COVID-19 transmission have reduced the availability of colonoscopies in most health system (1, 2, 15). This situation has complicated the chronic problem, present before the pandemic, of insufficient colonoscopy availability in many public health systems. Indeed, waiting lists for endoscopies (for patients with symptoms) and CRC screening have been the norm (16–18). Here, we evaluated two scenarios and options for coping with this problem in CRC screening programs that use FIT to select patients for diagnostic colonoscopies.

We proposed a simple and flexible methodology that can be extended to other sub-populations and even to other screening programs for decision-making, which becomes important in this or other situations.

Previous studies have evaluated the impact of raising the FIT cut-off point to match the demand to the availability of colonoscopies in CRC screening programs (4, 19). However, this option is not always followed in countries that use FIT as the screening test, because it implies that lesions with insufficient bleeding might not test positive, and thus, would be missed. To address that concern, in the present study, we evaluated two alternative scenarios for reducing the number of patients screened, and we provided a comparison of the proportions, types, and numbers of CRC and adenoma lesions that would be missed in each scenario.

Maintaining the cut-off point when the system cannot provide a sufficient number of colonoscopies leads to delays in diagnoses that could increase exponentially over the years. Thus, a large number of patients with neoplastic lesions would not be treated, until after a significant delay or when they become symptomatic. Delays can lead to lesion progression to cancer or advanced stages, and even death, which goes against the objective of screening (20). However, we found that the poor results of scenario 1, where the cut-off value was not modified, could be reduced by introducing additional factors into the selection process, such as the sex of the target population. We chose sex prioritization, because the proportion of serious and more advanced lesions was higher among men than among women. Age is another significant risk factor, and both these factors could be introduced to optimize the results of the screening program without modifying the cut-off point. Thus, in Scenario 1, fewer target patients were missed with prioritization than with the random criterion. In both cases, the magnitude and impact of the method on the numbers of undiagnosed lesions that accumulated over the years depended on how much the availability of colonoscopies was reduced, compared to the ideal number; nevertheless, the random criterion always had a greater impact than the prioritization criterion.

In contrast, raising the FIT cut-off point would not cause an accumulation of delayed colonoscopies. However, a number of lesions would not be diagnosed until the next round(s) or when the lesion becomes symptomatic. The advantage of this approach is that the program will cover the target population every 1–2 years, and no factors need to be introduced “a priori” to optimize the results. In contrast, maintaining the FIT cut-off point would significantly delay the screening of the whole target population. In comparing these strategies, our results showed that increasing the FIT cut-off point should be preferred, because the number of lesions missed was systematically lower than those missed by maintaining the FIT cut-off and delaying the screening.

Our study had some limitations. First, we focused on the 60–70-year-old population, because it was the population screened in our current regional program. This population probably had a higher number of lesions than individuals aged 50–60 years, who are typically included in most CRC screening programs. Nevertheless, we believe that the methodology and the main conclusions were valid; only the number of lesions missed or delayed might change in different populations.

Additionally, we assumed that the proportion of people that accepted the invitation to undergo a colonoscopy, and the proportion of lesions found in colonoscopies would remain stable over time. Clearly, this assumption might not be upheld, but with progressive implementation of the screening program, the level of participation and the outcomes should stabilize over time.

Increasing the cut-off of FIT will increase the number of interval cancers and the progression of some adenomas to cancer after negative FITs, especially if the increased cut-off level is maintained over time, which implies that our study may underestimate the effects of raising the cut-off. However, it is also true that the risk of detecting a colon cancer increases with the FIT value, and the effect may be higher for low-risk lesions (18). Also, CRC screening programs triggers the demand for surveillance colonoscopy, which will affect the colonoscopy capacity in the subsequent years. This effect has not been taken into consideration in our analysis since it is difficult to make realistic estimation.

In conclusion, we provided an evidence-based methodology that might facilitate decision-making in CRC screening programs, when the demand exceeds the availability of colonoscopies, under any potential circumstances, such as the current situation with the COVID-19 pandemic. Our results showed that, assuming a mismatch between the annual availability of colonoscopies and the actual demand in the target population, it is better to increase the cut-off point than to maintain the cut-off point and call people at random to undergo the colonoscopy. In addition, our findings argued for the inclusion of risk factors, like sex and probably age, in the selection process, to minimize the number of missed high-risk lesions.

## Data Availability Statement

The data analyzed in this study is subject to the following licenses/restrictions: Data sharing is not applicable to this article as no new data were created in this study. Requests to access these datasets should be directed to pcarreralasfuentes@gmail.com.

## Ethics Statement

This study was reviewed and approved by the Regional Ethical Committee of Aragón (CEICA). Written informed consent from the participants was not required since this study is retrospective and used data stored in databases, which were anonymized for data analysis.

## Author Contributions

ÁL and MD coordinated the whole work, reviewed the drafted manuscript, discussed the results, and approved the submitted manuscript. ÁL identified the clinical problem, defined the associated objectives, revised the clinical results, and got the data. MD supervised the technical approach and the corresponding results. RA-G and PC-L prepared the data and did the statistical analysis. Rd-H-A made the computations and graphics. ÁL, MD, and RA-G drafted the manuscript, while PC-L and Rd-H-A also contributed to the final version. ÁL and Rd-H-A obtained funding. All authors contributed to the article and approved the submitted version.

## Conflict of Interest

ÁL was advisors to Sysmex Iberica. Spain. The remaining authors declare that the research was conducted in the absence of any commercial or financial relationships that could be construed as a potential conflict of interest.

## Publisher's Note

All claims expressed in this article are solely those of the authors and do not necessarily represent those of their affiliated organizations, or those of the publisher, the editors and the reviewers. Any product that may be evaluated in this article, or claim that may be made by its manufacturer, is not guaranteed or endorsed by the publisher.

## Footnotes

^1^Available online at: https://www.cancer.org/cancer/colon-rectal-cancer/detection-diagnosis-staging/staged.html (accessed: Januray 29, 2021).
